# Viperin Targets Flavivirus Virulence by Inducing Assembly of Noninfectious Capsid Particles

**DOI:** 10.1128/JVI.01751-17

**Published:** 2017-12-14

**Authors:** Kirstin Vonderstein, Emma Nilsson, Philipp Hubel, Larsård Nygård Skalman, Arunkumar Upadhyay, Jenny Pasto, Andreas Pichlmair, Richard Lundmark, Anna K. Överby

**Affiliations:** a Department of Clinical Microbiology, Virology, Umeå University, Umeå, Sweden; b Laboratory for Molecular Infection Medicine Sweden, Umeå University, Umeå, Sweden; c Max Planck Institute for Biochemistry, Martinsried, Munich, Germany; d Department of Medical Biochemistry and Biophysics, Umeå University, Umeå, Sweden; e Integrative Medical Biology, Umeå University, Umeå, Sweden; University of Texas Southwestern Medical Center

**Keywords:** COPI, COPII, GBF1, assembly, capsid, flavivirus, interferon, tick-borne encephalitis virus, viperin

## Abstract

Efficient antiviral immunity requires interference with virus replication at multiple layers targeting diverse steps in the viral life cycle. We describe here a novel flavivirus inhibition mechanism that results in interferon-mediated obstruction of tick-borne encephalitis virus particle assembly and involves release of malfunctioning membrane-associated capsid (C) particles. This mechanism is controlled by the activity of the interferon-induced protein viperin, a broad-spectrum antiviral interferon-stimulated gene. Through analysis of the viperin-interactome, we identified the Golgi brefeldin A-resistant guanine nucleotide exchange factor 1 (GBF1) as the cellular protein targeted by viperin. Viperin-induced antiviral activity, as well as C-particle release, was stimulated by GBF1 inhibition and knockdown and reduced by elevated levels of GBF1. Our results suggest that viperin targets flavivirus virulence by inducing the secretion of unproductive noninfectious virus particles via a GBF1-dependent mechanism. This as-yet-undescribed antiviral mechanism allows potential therapeutic intervention.

**IMPORTANCE** The interferon response can target viral infection on almost every level; however, very little is known about the interference of flavivirus assembly. We show here that interferon, through the action of viperin, can disturb the assembly of tick-borne encephalitis virus. The viperin protein is highly induced after viral infection and exhibit broad-spectrum antiviral activity. However, the mechanism of action is still elusive and appears to vary between the different viruses, indicating that cellular targets utilized by several viruses might be involved. In this study, we show that viperin induces capsid particle release by interacting and inhibiting the function of the cellular protein Golgi brefeldin A-resistant guanine nucleotide exchange factor 1 (GBF1). GBF1 is a key protein in the cellular secretory pathway and is essential in the life cycle of many viruses, also targeted by viperin, implicating GBF1 as a novel putative drug target.

## INTRODUCTION

The type I interferon (IFN) system is the first line of antiviral defense and an important part of the intrinsic innate immune response that controls virus dissemination and protects against serious disease. Binding of IFN to the IFN receptor activates a signaling cascade that leads to the transcriptional activation of hundreds of IFN-stimulated genes (ISGs), which encode proteins with diverse biological function where some are potent antiviral proteins and part of the response against mammalian viruses (1). The antiviral function of ISGs is only partially understood. However, it is well accepted that ISGs target different steps in the virus life cycle ranging from cell entry, virus protein translation, genome replication, and exit of virus particles (reviewed in reference 2).

The genus Flavivirus, within the family of Flaviviridae, comprises important human pathogens transmitted by mosquitos and ticks, such as yellow fever virus, dengue virus (DENV), West Nile virus, Zika virus, and tick-borne encephalitis virus (TBEV). These are small (∼50-nm), spherical, enveloped, positive-stranded RNA viruses. It is well known that the type I IFN response is crucial for restricting different flaviviruses, and several ISGs have been identified to restrict flavivirus growth. However, very little is known about how specific ISGs target the assembly and release of virions. Prior to the assembly of virions, a single polyprotein is translated from the viral RNA genome. The polyprotein is cleaved into seven nonstructural proteins and three structural proteins. Although the nonstructural proteins promote genomic replication and assembly, the three structural proteins—capsid (C), prM/M (membrane and its precursor), and envelope (E)—form the viral particles (3). The C proteins of all flaviviruses are highly basic, which gives them the ability to bind the viral genomic RNA to form a nucleocapsid, which buds into the endoplasmic reticulum (ER), acquiring a host-derived lipid bilayer coated by the membrane-bound glycoproteins prM and E (4–6). From the ER, the virion is transported along the secretory pathway toward the Golgi compartment, where the maturation of carbohydrate groups on prM and E, as well as the cleavage of prM to M, occurs (7). Finally, the mature virion is released by exocytosis (reviewed in reference 3).

Two major ISGs, tetherin and viperin (virus inhibitory protein, ER associated, IFN inducible), have been shown to affect assembly and release of viruses (reviewed in reference 2). Tetherin is encoded by the ISG *BST2* and is localized within lipid rafts on the cell surface, in the *trans*-Golgi compartment, and/or within recycling endosomes (8, 9). It inhibits release of human immunodeficiency virus type 1 (HIV-1) viral particles by anchoring the virion to the plasma membrane (10), leading to internalization and degradation (11, 12). Viperin, encoded by the ISG *RSAD2*, is highly induced in an IFN-dependent or -independent manner (as reviewed previously [13]). Viperin is located to the cytoplasmic side of the ER (14) and has a broad-spectrum antiviral activity against many different enveloped viruses, e.g., DENV, West Nile virus, TBEV, hepatitis C virus (HCV), HIV-1, influenza A virus, Sindbis virus, Chikungunya virus, and human cytomegalovirus (13, 15–24). Viperin interferes with HIV-1 and influenza A virus budding from the plasma membrane. It binds and inhibits farnesyl diphosphate synthase (FPPS), an enzyme involved in isoprenoid biosynthesis, leading to altered fluidity of lipid rafts, thereby interfering with virus budding (21, 22). Viperin also inhibits genome replication of DENV and HCV by interacting with viral nonstructural proteins (15, 16, 23). The antiviral mechanism(s) of action are poorly understood for most viruses and seem to be dependent on the virus. However, viral or cellular proteins important in the viral life cycle are often sequestered by viperin (21, 23, 25).

In this study, we show that type I IFN treatment interferes with the assembly of TBEV virions. We identified that the viperin protein is responsible for this effect and demonstrate that viperin interacts with and inhibits the function of the GBF1 (Golgi brefeldin A-resistant guanine exchange factor 1) protein, a key factor for the secretory pathway. This interaction affects the assembly of progeny virions by strongly increasing the release of enveloped malfunctioning particles, thereby reducing the production of infectious particles.

## RESULTS

### IFN treatment induces secretion of capsid protein.

**FIG 1:**
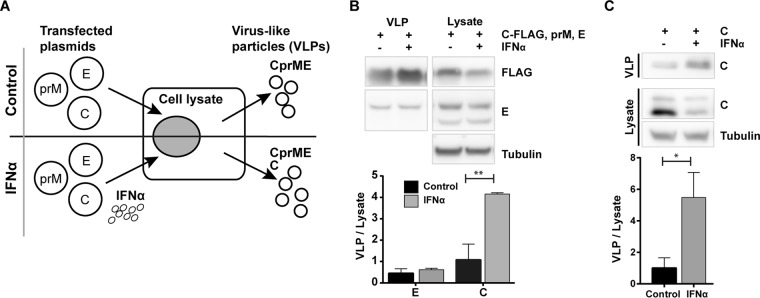
IFN-α induces the release of C protein from cells. (A) Schematic drawing of the experimental setup. (B) Release of CprME-VLPs in the presence or absence of IFN-αB/D. A549 cells were transfected with plasmids expressing TBEV Hypr C-FLAG, prM, and E. At 8 h posttransfection, 10,000 U/ml IFN-αB/D was added, and the supernatant and cell lysate were harvested 48 h later. Supernatants were concentrated by ultracentrifugation, and proteins were separated and detected using immunoblotting with the indicated antibodies. (C) Release of C protein in the presence of IFN-αB/D. A549 cells were transfected with only C protein expression plasmid and treated as described in panel B, except that C proteins were detected with rabbit anti-C antibody. Representative blots are shown; graphs show quantification (means and standard deviations [*n* = 3]) of the Western blots, where C and E proteins in the supernatant were normalized to proteins C and E in the lysate. **, *P* < 0.01; *, *P* < 0.05 (Student *t* test).

IFN and the expression of ISGs can target almost any step of the viral life cycle. However, very little is known about the effect of IFN on particle assembly and egress of flaviviruses. To study viral particle release, we established an expression system that allows the generation of virus-like particles (VLPs) composed of the structural proteins C, prM, and E (Fig. 1A) (26). This system enabled us to study the secretion of VLPs into the supernatant of transfected A549 cells (Fig. 1B). To test the influence of IFN-α, we compared intracellular and extracellular abundance of flavivirus protein and particles in presence or absence of recombinant IFN-αB/D (Fig. 1B). The effect of IFN treatment on particle assembly was measured by quantifying the ratio between secreted E and C proteins (VLP) and E and C proteins in the cell lysate (Lysate). Surprisingly, compared to control treatment, IFN-αB/D treatment led to a strong increase in C protein release, while the secretion of E protein remained unaltered (Fig. 1B). Flavivirus C protein has not previously been described to be secreted separately from the other structural proteins. To test this, only TBEV C protein was expressed in A549 cells and found to be sufficient for protein release (Fig. 1C). Again, treatment of transfected cells with IFN-αB/D led to an increase of C protein secreted to the supernatant (Fig. 1C). Collectively, this suggested that type I IFN affect the viral structural proteins differently, indicating that C and E proteins are secreted by different mechanisms.

### Capsid particles are membrane associated and exit the ER via a COPII dependent mechanism bypassing the Golgi compartment.

**FIG 2:**
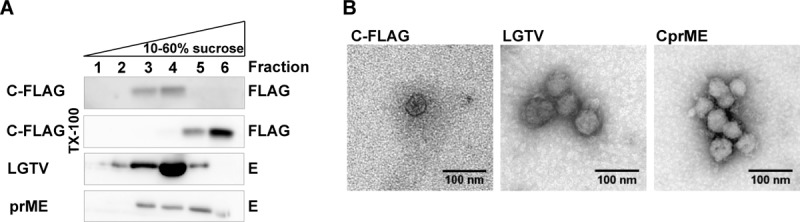
C protein is released as membrane-associated particles. (A) A flotation assay was used to determine the membrane association of C protein, prME VLPs, or LGTV. Supernatant from HeLa cells, which were transiently transfected with plasmids expressing C-FLAG or prM and E, and VeroB4 cells infected with LGTV were concentrated by ultracentrifugation, followed by a flotation assay. The gradient was fractionated from top to bottom and analyzed by immunoblot analysis. Representative blots from two independent experiments are shown. (B) Morphology of VLPs and LGTV as determined by transmission electron microscopy. Supernatant from cells transfected with C-FLAG or the structural proteins C, prM, and E or infected with LGTV were harvested, concentrated by using a flotation assay, and analyzed by negative staining after glutaraldehyde fixation. Representative images are shown.

Since flavivirus C protein secretion has not been described before, we set out to characterize the phenomenon in detail. The C protein detected in the supernatant could be released from cells as soluble proteins, protein aggregates, or membrane-associated proteins. To characterize the nature of the secreted C protein, a flotation assay was performed. C protein floated up and behaved (Fig. 2A, first row) in the same manner as Langat virus (LGTV; a low-virulence member of the TBEV serogroup, which has been used extensively as a nonpathogenic models for TBEV) and VLPs containing prM and E (Fig. 2A). This showed that the C protein released from cells was membrane associated. After the addition of detergent, the C protein was mainly found in the pellet (Fig. 2A, second row, fraction 6), confirming the membrane association. Analysis of the enriched C-particle fraction using electron microscopy revealed particles with a round morphology similar to LGTV and VLPs containing C, prM, and E (Fig. 2B).

**FIG 3:**
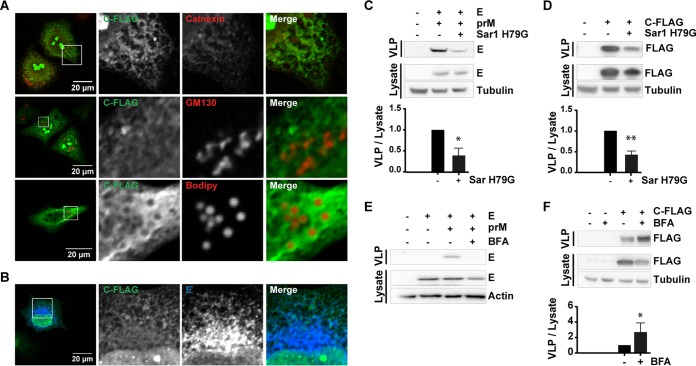
E and C localize to the ER and prME VLP release is COPII and COPI dependent, whereas C-particle release is only COPII dependent. Intracellular localization of C-FLAG with different cellular markers (A). HeLa cells were transfected with C-FLAG, fixed, and stained using antibodies against FLAG, calnexin (ER), and GM130 (Golgi). Lipid droplets were stained with Bodipy 493/503 at 1 μg/ml. For a clear arrangement, the lipid droplets are colored red in the overlay. (B) HeLa cells transfected with C-FLAG, prM, and E protein were stained with antibodies against FLAG and TBEV E. (C) Secretion of prME protein was analyzed in HeLa cells transfected with or without Sar1 H79G (dominant-active mutant) by immunoblot analysis. (D) Immunoblot analysis showing the intracellular expression of C and the secreted C in the presence or absence of Sar1 H79G. (E and F) Involvement of COPI during prME- or C-particle release. Immunoblot analysis showing the intracellular expression of E and C, and also E and C secreted from transfected HeLa cells treated with 0.5 μg/ml BFA (E and F, respectively). Representative blots are shown; the graphs show the quantification (means and standard deviations from *n* = 3 [C, D, and E] and *n* = 4 [F] Western blot experiments), where protein in the supernatant (VLP) was normalized to protein in the lysate. Significance was calculated using a Student *t* test (*, *P* < 0.05; **, *P* < 0.01).

It is generally assumed that flavivirus virions assemble in the ER and exit via the conventional secretory pathway (7, 27), and lipid droplets have been suggested to play a role in DENV encapsidation (28). However, to gain further information on the exact localization of TBEV C protein, we performed confocal analysis of cells transfected with plasmid encoding C-FLAG. The C protein was found in the nucleus and colocalized with E and the ER marker calnexin (ER) but not with GM130 (Golgi matrix protein) and was only in some cells associated with lipid droplets (Fig. 3A and B). To specifically block COPII-mediated transport from the ER toward the ERGIC (ER-Golgi intermediate compartment) and Golgi compartment, a dominant-active mutant of the small GTPase Sar1 (Sar1-H79G) was overexpressed, together with prME or C, and progeny particles were monitored. The Sar1 mutant binds to the ER and initiate the COPII coat protein recruitment but is not able to detach from the ER, thereby blocking ER-to-Golgi-compartment transport (29). The overexpression of Sar1-H79G blocked both prME-particle and C-particle release (Fig. 3C and D), suggesting that COPII-mediated anterograde transport is involved in the release of C particles. Next, the cells were treated with brefeldin A (BFA), which blocks both retrograde transport via COPI vesicles and the secretion from the *trans*-Golgi compartment via clathrin-coated vesicles and subsequently causes disruption of the Golgi apparatus (30). This leads to a collapse of the Golgi compartment into the ER and thereby blocks the release of proteins via this pathway (31). BFA efficiently inhibited the secretion of prME particles (Fig. 3E). However, BFA increased the secretion of C particles similar to the IFN treatment (Fig. 3F and Fig. 1C).

Our data suggest that C protein is released as membrane-associated particles and transported from the ER via a COPII-dependent mechanism. However, whereas virus particles and prME VLPs use the classical COPI-dependent secretory pathway, the C particles are released from the cell via a different mechanism, which is induced by both BFA and IFN treatment.

### Viperin enhances the release of TBEV capsid particles.

**FIG 4:**
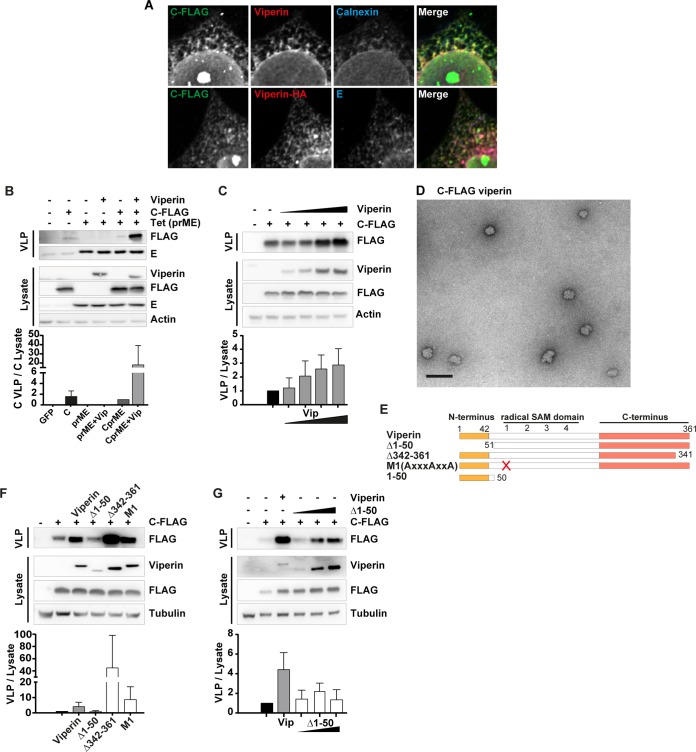
Viperin colocalizes with C and E and induces C-particle release via its N-terminal domain. (A) HeLa cells transfected with C-FLAG and viperin or with C-FLAG, viperin-HA, prM, and E protein stained with antibodies against FLAG, HA, viperin, calnexin, and TBEV E. Representative confocal images are shown. (B) Release of CprME-VLPs in the presence or absence of viperin. A 293 FLP-IN T Rex cell line inducibly expressing TBEV Hypr prME was treated with 1 μg/ml tet and transiently transfected with wt viperin and TBE Hypr C 3× FLAG (C-FLAG). Western blot analysis showing the intracellular and secreted proteins. (C) Dose-dependent release of C protein in the presence of viperin. HeLa cells were transfected with C-FLAG and increasing amounts of viperin (0.75, 1.5, 3, and 6 μg). (D) Morphology of C-FLAG particles by transmission electron microscopy. Supernatants from cells transfected with C-FLAG and viperin were harvested, concentrated using a flotation assay, and analyzed by negative staining after glutaraldehyde fixation. Scale bar, 100 nm. A representative picture is shown. (E) Schematic drawing of viperin mutants. (F) Secretion of C protein in the presence of wt and viperin mutants. HeLa cells were transfected with wt, Δ1-50, Δ341-361, and mutant M1, together with plasmid encoding C–FLAG. (G) Dose-independent release of C protein in the presence of mutant Δ1-50. Increasing amounts of plasmid expressing Δ1-50 were transfected into HeLa cells, together with C-FLAG. C protein release was measured with or without wt viperin expression. Representative blots are shown; graphs show the quantification (means and standard deviations from *n* = 3 [B and C] and *n* = 2 [F and G] Western blot experiments), where C in the supernatant is normalized to C in the lysate.

To our knowledge, an ISG that positively regulates and induce budding of particles has not been reported. TBEV is budding into the ER and a prominent ISG that localizes to this compartment is viperin. Viperin has previously been reported to inhibit assembly and egress of influenza A and HIV-1 (21, 22) and inhibits TBEV replication (19). Interestingly, the effect on TBEV particle release is greater than the effect on RNA replication (19), suggesting a viperin activity on posttranscriptional level. Viperin colocalized with calnexin (Fig. 4A), confirming it is localization to the ER. Moreover, we could colocalize viperin with C and E protein (Fig. 4A), suggesting an involvement of viperin in particle formation.

To assay the effect of viperin on virus protein secretion, we cotransfected FLAG-tagged C and viperin into 293 FLP-IN T Rex cells expressing prME upon addition of tetracycline (tet). The accumulation of secreted proteins in the supernatant was analyzed by immunoblotting for E protein and C-FLAG, respectively. Expression of viperin did not have an effect on TBEV prME-particle secretion (Fig. 4B) or NS1 protein secretion (data not shown). However, the amount of released C protein increased drastically in the presence of viperin (Fig. 4B). The positive effect on C protein release depended on the dose of transfected viperin (Fig. 4C). In agreement with this, C particles were detected in the supernatant by electron microscopy when C-FLAG and viperin were coexpressed (Fig. 4D).

Next, we analyzed which domains in viperin were involved in the enhanced release of C, using transient transfection of different mutated versions of viperin (Fig. 4E). Truncation in the C terminus (Δ342-361) and the mutant in the radical SAM motif (M1) did not impair the ability of viperin to induce secretion of C protein (Fig. 4F). However, deletion of the N-terminal amphipathic alpha-helix (Δ1-50, also known as TN50 [19]), which directs the protein to the ER (14) significantly reduced the potency of viperin to promote C protein release (Fig. 4F and G).

**FIG 5:**
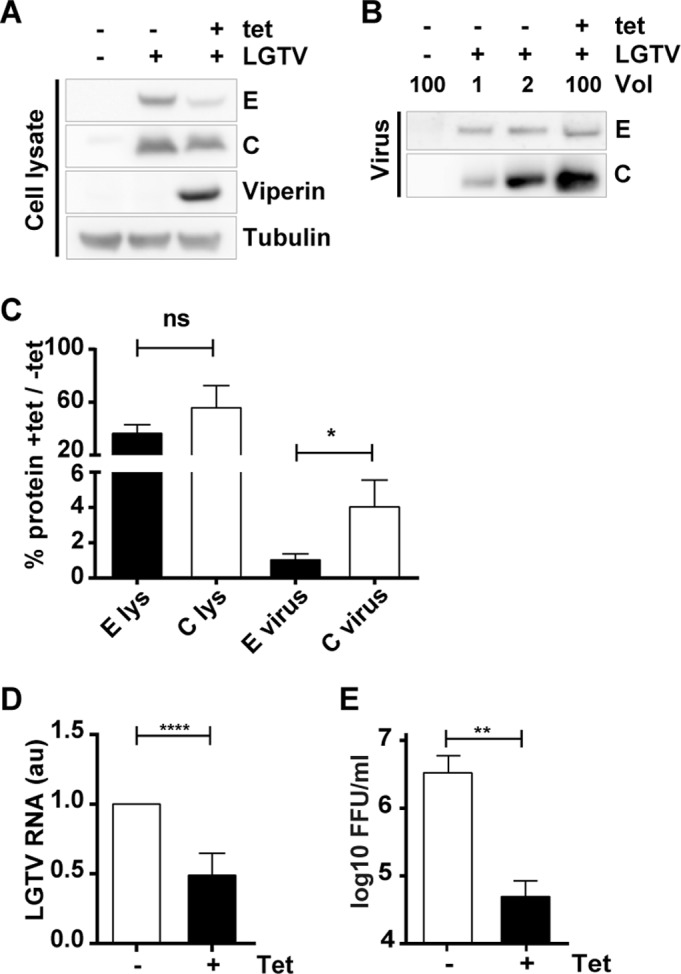
Viperin induces LGTV C protein secretion during infection. 293 FLP-IN T Rex cells inducibly expressing viperin were infected with LGTV and either treated with 1 μg/ml tet at the time point of infection or left untreated. Cells and virus in the supernatant were harvested 48 h postinfection. (A) Amounts of viral proteins E and C detected in cell lysate by Western blotting. (B) Supernatants were concentrated by ultracentrifugation, and comparable levels of viral E proteins with or without viperin were loaded and detected by Western blotting. The lower panel shows the corresponding C protein in the supernatant. Representative blots are shown from three independent experiments. (C) Percentages of E and C protein in the presence of tet-induced viperin compared to uninduced quantification (means and standard deviations) in Western blots of E and C from the lysate (A) and released particles (B) from three independent experiments. (D) LGTV RNA levels in the cell lysate measured by real-time RT-PCR. (E) Plaque assay detecting the infectivity of progeny virions. Mean values and standard deviations (*n* = 6 [D and E]). Significance was calculated with a Student *t* test (*, *P* = 0.0275; **, *P* < 0.01; ****, *P* < 0.0001).

To verify the antiviral effect of viperin on particle release during viral infection, 293 FLP-IN T Rex cells inducibly expressing viperin upon addition of tet were infected with a high multiplicity of LGTV. Viperin showed a clear antiviral activity against LGTV (Fig. 5), and the antiviral effect was stronger on released viral particles (Fig. 5B) and infectivity (Fig. 5E) than on the viral RNA (Fig. 5D) and viral proteins in the cell lysate (Fig. 5A). The total amount of C protein in the supernatant compared to the E protein (Fig. 5B and C) was greatly enhanced in the presence of viperin (Fig. 5C).

Collectively, our data show that viperin induces C protein release both during viral infection and in transient transfected cells expressing C protein. This function requires localization to the ER and does not impair other cellular secretion systems.

### Viperin interacts with GBF1 and induce C-particle secretion.

**FIG 6:**
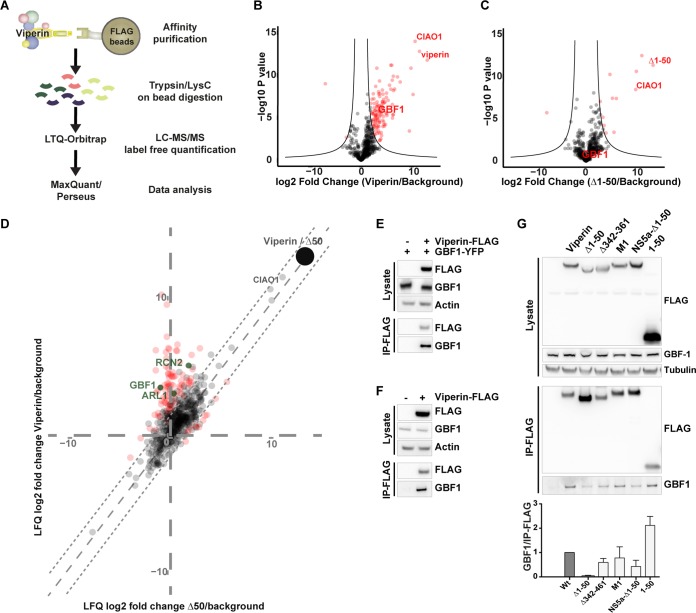
Identification of the protein interactome of viperin by mass spectrometry analysis and verification of GBF1. (A) Schematic representation of an affinity purification/MS strategy. N-terminal FLAG-tagged viperin, Δ1-50 mutant, and GFP (as control) were expressed by tet treatment of 293 FLP-IN T-Rex cells that contained a stably integrated transgene or not expressed when tet was omitted. Cells were lysed under mild lysis conditions, and proteins were precipitated using FLAG beads. After precipitation and extensive washing, the proteins were digested with trypsin and LysC, and the peptides were purified and analyzed by LC-MS/MS. (B and C) Volcano blots of proteins enriched in viperin wt (B) or Δ1-50 (C) precipitates compared to the GFP background control. The hyperbolic line delineates nonsignificantly to significantly enriched proteins. (D) Two-dimensional scatter plot comparing proteins enriched in viperin or the Δ1-50 mutant. Colors: red, significant changes versus the background (two-tailed Welch's *t* test [cutoff: FDR = 0.001 with S0 = 1]); green, significant candidates with association with ER or the Golgi compartment. (E and F) Coimmunoprecipitation analysis shows the interaction between viperin and GBF1. FLAG-tagged viperin was immunoprecipitated with anti-FLAG antibody either from extracts of HEK293T cells transfected with FLAG-tagged viperin and YFP-tagged GBF1 (E) or only FLAG-tagged viperin (F). (G) Coimmunoprecipitation analysis of endogenous GBF1 and viperin mutants. HEK293T cells were transfected with plasmids expressing FLAG-tagged wt viperin, Δ1-50, Δ341-361, M1, HCV NS5a-Δ1-50, or 1-50 mutant and immunoprecipitated with FLAG antibody. HCV NS5a-Δ1-50 is a chimera with the HCV-NS5a amphipathic alpha helix fused to the viperin Δ1-50 mutant (19). Immunoblots show protein input, and immunoprecipitation (IP) graphs show the quantification of IP GBF1 (means and standard deviations from three independent experiments).

Next, we aimed to reveal the mechanism behind viperin-induced secretion of C particles. As no direct interaction between viperin and C protein could be detected (data not shown), host factors might be involved. We therefore used an affinity proteomics approach to study cellular interaction partners of viperin. We precipitated wild-type (wt) viperin, the Δ1-50 viperin mutant, or GFP as a control and analyzed associated proteins by liquid chromatography coupled to tandem mass spectrometry (LC-MS/MS) (Fig. 6A).

We identified 115 proteins specifically binding to viperin or the Δ1-50 mutant (Fig. 6B and C; see also Table S1 in the supplemental material). Direct comparison of viperin to the Δ1-50 mutant identified 34 proteins that were significantly more enriched in the wt protein precipitates compared to precipitations with the mutant protein (Fig. 6D, red and green dots). Ciao1 identified previously (19) is comparably well enriched by wt viperin and Δ1-50 mutant, suggesting that the immunoprecipitation (IP) conditions were similar overall. As additional filter method we considered the subcellular localization of these proteins using annotations based on the Human Protein Atlas. This analysis showed that the majority proportion of candidates localized to the nucleus. However, three proteins ARL1 (ADP-ribosylation factor-like protein 1), RCN2 (ERC-55; ER Ca2^+^ binding protein, 55 kDa), and GBF1 (Fig. 6D, green dots) were annotated to localize to the ER or the Golgi compartment. Of these proteins, ARL1 has been reported to regulate intracellular trafficking between the plasma membrane, endosomes, and the *trans*-Golgi compartment (32). RCN2 has been shown to be a chaperone in the ER and involved in signal transduction (33). GBF1 is a GTP-exchange factor (GEF), which is involved in COPI trafficking. BFA treatment is known to inhibit COPI-coated vesicle formation by binding to and stabilizing the GBF1-ARF1-GDP complex (34). Since BFA induces C-particle secretion (Fig. 3F), the positive effect of viperin on C-particle release could be explained by GBF1 targeting. We therefore set out to further study the interaction between viperin and GBF1. The interaction between viperin and GBF1 could be confirmed by coimmunoprecipitation of both transiently transfected and of endogenous GBF1 (Fig. 6E and F). Mapping of the interaction domain revealed that the 50 first amino acids of viperin containing the amphipathic alpha-helix were important for the binding GBF1 (Fig. 6G), further confirming the mass spectrometry data.

**FIG 7:**
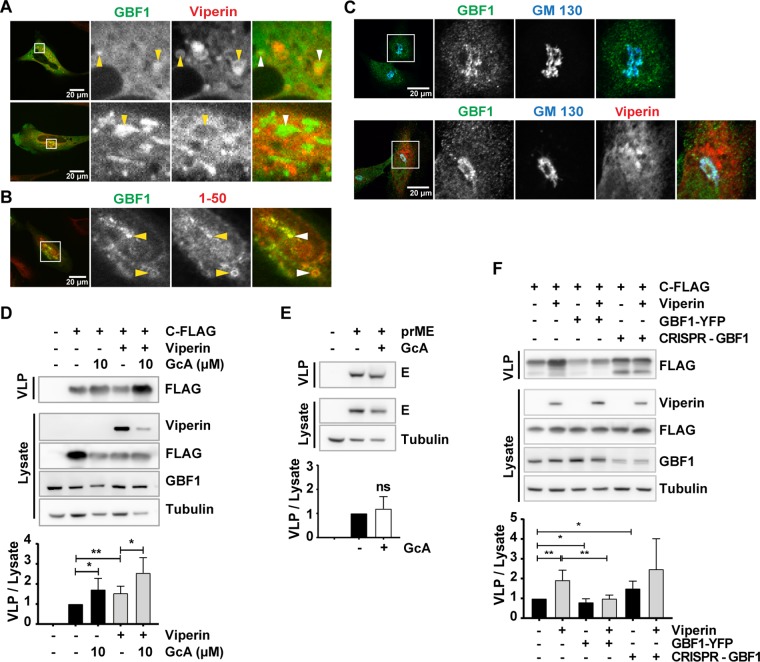
Viperin sequester GBF1 to induce C-particle release. (A) HeLa cells transiently expressing eGFP-GBF1 and mCherry-viperin analyzed in Live-cell confocal spinning disc microscopy show the colocalization of GBF1 and viperin. Arrowheads indicate vesicular structures. (B) Colocalization of the overexpression of viperin 1-50 and eGFP-GBF1 in HeLa cells. (C) Overexpression of mCherry-viperin in HeLa cells and localization of endogenous GBF1 relative to the Golgi marker GM130. Representative confocal images of nontransfected control cells (upper row) and cells transfected with mCherry-viperin (lower row) are shown. The involvement of GBF1 during C (D)- or prME (E)-particle release was assessed. Immunoblot analysis shows the intracellular expression of C (in the presence or absence of viperin) and E, as well as the secreted C and E in transfected HeLa cells treated with 10 μM Golgicide (GcA), a specific GBF1 inhibitor. (F) Effect of availability of wt GBF1 on the release of C protein. HeLa cells were transfected with GBF1-YFP to increase cellular amounts or with CRISPR Cas9 GBF1 plasmid to knock down the cellular GBF1 level. The amounts of protein in the supernatant and cell lysate were measured by immunoblotting at 48 h after the second transfection. Representative blots are shown; graph show the quantification (means and standard deviations from a minimum of *n* = 4 Western blot experiments [D to F]), where C or E in the supernatant is normalized to C and E in the lysate. Significance was calculated using a Student *t* test (*, *P* < 0.05; **, *P* < 0.01).

To gain additional knowledge on the subcellular localization of viperin and GBF1, we assessed the subcellular localization of both proteins. Live cell imaging analysis of mCherry-viperin and eGFP-GBF1 showed that eGFP-GBF1 localized to vesicular structures surrounded by viperin (Fig. 7A). The colocalization between GBF1 and the N-terminal 1 to 50 amino acids of viperin in such structures was even more pronounced (Fig. 7B), confirming the coimmunoprecipitation results and suggesting a critical role of the viperin N terminus in these assays. The steady-state distribution of GBF1, together with the Golgi marker GM130, was not altered in the presence of viperin (Fig. 7C). To clarify whether the function of GBF1 is involved in C-particle secretion, we used a specific GBF1 inhibitor, Golgicide. Cells transfected with plasmids expressing C-FLAG and/or viperin were treated with Golgicide, and the effect on C protein release was evaluated by immunoblotting as described before. Notably, the inhibition of GBF1 by Golgicide strongly induced secretion of C particles, especially in the presence of viperin (Fig. 7D). Importantly, no effect of Golgicide was detected on prME secretion (Fig. 7E), confirming a selective function of GBF1 in flavivirus protein release. To test the functional relationship between viperin and GBF1, the production of C particles was analyzed during modulated cellular levels of GBF1 and in the presence of viperin (Fig. 7F). Viperin-induced increase of C-particle release could be inhibited by overexpression of YFP-GBF1, and transient knockdown of GBF1 with CRISPR Cas9 induced the C-particle release (Fig. 7F), confirming a functional interaction between viperin and GBF1 and, in addition, the importance of GBF1 in the assembly of TBEV.

Taken together, our data demonstrate that viperin inhibits the function of GBF1, leading to the selective release of C particles. This decreases the total number of infectious TBEV particles and thereby reduces the overall infectivity.

## DISCUSSION

Very few studies have focused on the antiviral action of ISGs targeting the assembly of virions. Here, we show that type I IFN interferes with flavivirus assembly by inducing unproductive capsid particle release. We found that the IFN-stimulated protein viperin mediates this host cell response by interacting and interfering with the cellular protein GBF1, which is a central molecule in vesicle budding and remodeling of membranes. GBF1 has previously been shown to play a central role in the life cycle of many RNA viruses, which utilize vesicular trafficking for targeting of viral proteins, and cellular membranes in their replication cycle and assembly process (35–39). We found that interfering with GBF1 induce egress of membrane associated C particles, while leaving secretion of prME particles unaffected.

Particle assembly of flavivirus virions occurs by nucleocapsid budding into the ER acquiring the E and prM envelope near the replication complex (6, 40, 41). Secretion of both C particles and prME VLPs were found to be dependent on the conventional COPII secretory pathway, whereas only prME VLPs appeared be released via the Golgi compartment through conventional secretion, in a way similar to that described for other flaviviruses (7). C particles, however, are released from the cell via a different mechanism, which is induced by BFA, Golgicide treatment, and viperin expression.

Both viperin and GBF1 are able to peripherally attach to membranes of the ER, and viperin seems to affect protein secretion when membrane associated. Viperin has been shown to target the ER membrane via its N-terminal amphipathic helical domain (14, 19). This domain has also been found to induce crystalloid ER and thereby rearranging the smooth ER membranes into a lattice-like pattern (14). We demonstrate here that the enhanced secretion of C protein by viperin was dependent on its N terminus and binding to GBF1. Interestingly, the N-terminal domain of viperin seems to be very important for viperin function since the numbers of cellular proteins interacting with the mutant lacking the amphipathic helix were very low (see Table S1 in the supplemental material). This indicates that the intracellular localization of viperin to the ER membrane or the N-terminal itself is important for protein-protein interaction. This domain in viperin is also important for the inhibition of Chikungunya virus (18), TBEV (19), and HCV (16) and directly mediates the binding to GBF1. GBF1 normally regulates membrane dynamics in the secretory pathway, and most RNA viruses rely on cellular vesicular trafficking for proper intracellular localization of viral proteins. GBF1 is important in the life cycles of several different viruses: GBF1 is necessary for RNA replication of DENV, HCV, and SARS coronavirus (35–37), for particle assembly of Ebola virus and influenza A virus (38, 39), and for targeting of DENV C protein to lipid droplets, which is important for DENV infection (36, 42). An interaction between DENV C protein and the N-terminal of viperin has been shown (15). This interaction occurred at the interface of lipid droplets (15), a hub for DENV assembly (28). Interestingly, a physical interaction between viperin and TBEV C protein could not be detected and thus might not be necessary for the induced secretion of C particles. Immunofluorescence analysis revealed that the N-terminal region of viperin localized to vesicular structures, together with GBF1 in agreement that this region is sufficient to target GBF1. It can be envisaged that modulation of GBF1 by viperin would potentially modulate all of the above-mentioned functions both in the presence and in the absence of interaction between viperin and viral proteins and therefore constitute a widely used mechanism to impair virus spread.

Viperin increases the secretion of membrane-associated C protein but does not affect the transport and release of transmembrane protein prME or vesicular stomatitis virus glycoprotein (14). Similarly, inhibiting GBF1 with BFA completely blocks secretion of prME VLPs and soluble proteins (43) but induces the secretion of TBEV C particles, indicating that proteins are selectively loaded in transport vesicles and viperin affects protein secretion differently, depending on whether they are soluble, membrane-associated, or transmembrane proteins. This selectivity also suggests that cells expressing viperin can reduce viral assembly specifically while leaving the cytokine release untouched.

The membrane association of C protein observed in this study is probably mediated via a hydrophobic domain within the C protein. A similar type of hydrophobic membrane association of the C protein has been suggested for DENV, yellow fever virus, and West Nile virus (44, 45) and even tick-borne flaviviruses (LGTV, Powassan, and TBEV). Notably, the C protein appears to have an intrinsic ability *in vitro* to assemble into particles even in the absence of membranes (46, 47). However, assembly and release of capsid particle in cell culture have not been previously reported for flaviviruses. Interestingly, naked capsid-like HCV particles have been reported *in vivo* (48–50), raising the possibility that viperin might be involved in inducing secretion of such capsid-like HCV particles. We found that the membrane association of the TBEV capsid particles was quite sensitive to mechanical stress; therefore, it could be that the HCV C particles are also secreted with a membrane that is lost shortly after cellular release. HCV C particles are taken up by clathrin-mediated endocytosis in human hepatoma cells (48, 51) and can induce an immune response by modifying specific phenotypic and functional markers in T cells (52). TBEV C particles may have a similar immune modular capacity. A potential antiviral purpose of C-particle secretion could be multifarious ranging from being a side product of disturbed viral assembly, thereby reducing the overall infectivity, to having paracrine or endocrine functions, as reported for HCV (48, 52).

Taken together, we show that IFN selectively induces TBEV C-particle release from cells and that the ISG viperin expression increases C-particle release after infection. We identified GBF1 as a novel interaction partner to viperin. The mechanism behind the induced release of C particles seems to depend on the ability of viperin to interact with GBF1. Viperin is known to have an antiviral effect against a broad range of viruses such as DENV, HCV, influenza A virus, and Chikungunya virus (15, 18, 21, 25). Most of these viruses use GBF1 for their life cycles (35, 39, 53), suggesting that the viperin-GBF1 interaction might be relevant for the antiviral defense against many different viruses and identifying GFB1 as a novel putative drug target for antivirals.

## MATERIALS AND METHODS

### Cells, viruses, and reagents.

Simian Vero B4 cells were grown in M199 (Invitrogen), and human lung carcinoma cells (A549), HeLa cells, and HEK293T cells were grown in Dulbecco modified Eagle medium (DMEM), both supplemented with 5% fetal calf serum (FCS) and penicillin-streptomycin. Human 293 FLP-IN T Rex cells inducibly expressing wt viperin were kindly provided by Ju-Tao Guo. Human 293 FLP-IN T Rex cells (Invitrogen) inducibly expressing TBEV Hypr E and prM protein were generated according to protocol (Invitrogen). Human 293 FLP-IN T Rex cells were propagated in DMEM supplemented with 5% tetracycline-negative FCS (PAA) and penicillin-streptomycin. For induction, 1 μg/ml tetracycline (Sigma) was used. Langat strain TP21 (kindly provided by Gerhard Dobler, Bundeswehr Institute of Microbiology, Munich, Germany) was propagated in Vero B4 cells under biosafety laboratory 2 conditions. BFA stock solution was 5 mg/ml in ethanol (Sigma). Golgicide (Sigma) stock solution was 17 mM in dimethyl sulfoxide. The recombinant human IFN-αB/D hybrid (54) (kindly provided by Peter Stäheli, Institute of Virology, Medical Center University of Freiburg, Freiburg, Germany).

### Plasmids.

Expression plasmids encoding TBE Hypr C, C 3× FLAG, prM, and E (41)—as well as human wt viperin, Δ1-50 (TN50), Δ342-461 (TC20), and M1 mutant viperin—and N-terminally FLAG-tagged wt and mutated viperin in the eukaryotic expression vector pI.18 or pcDNA 3.1 have all been described previously (19). The YFP-GBF1-wt plasmid (34) was kindly provided by Catherine L. Jackson, Institut Jacques Monod, University Paris Diderot, Paris, France. The eGFP-GBF1-wt plasmid was generated from the YFP-GBF-wt plasmid by site-directed mutagenesis. The pIRES DsRed2 Sar1 H79G (active mutant) plasmid (55) was kindly provided by Hirofumi Kai, Kumamoto University, Kumamoto, Japan. The mCherry-viperin was constructed by lifting viperin from pI.18 into a mCherry backbone. Transfection was performed with Nanofectin (PAA) or GeneJuice (Novagen) according to the manufacturer's protocol; the transfection efficiency ranged between 40 and 80%.

### Antibodies.

Primary antibodies were directed against TBEV E (mouse monoclonal antibodies 1493.1 and 1786.3 [56]). TBEV C protein polyclonal antibody was generated in rabbits by immunization with peptide CMVKKAILKGKGGGPPRRVSK according to a standard protocol (Agrisera). Additional primary antibodies included the following: actin (rabbit polyclonal; Sigma), FLAG epitope (mouse monoclonal M2 [Stratagene]; chicken polyclonal [Abcam]; and rabbit polyclonal [Sigma]), HA epitope (rabbit polyclonal; Abcam), viperin (rabbit polyclonal and mouse monoclonal; Abcam), calnexin (rabbit polyclonal and mouse monoclonal; Abcam), GM130 (mouse monoclonal; BD Biosciences), beta-tubulin (rabbit polyclonal; Abcam), eGFP (rabbit polyclonal; Invitrogen), and GBF1 (rabbit polyclonal [Abcam], rabbit polyclonal [Invitrogen]). Secondary antibodies included goat anti-chicken Alexa Fluor 488 (Invitrogen), goat anti-chicken Alexa Fluor 555 (Abcam), donkey anti-mouse Alexa Fluor 488/555 and donkey anti-rabbit Alexa Fluor 488/555 (Invitrogen), goat anti-mouse Alexa Fluor 647 (Life Technologies), goat anti-rabbit Alexa Fluor 488 (Life Technologies), and horseradish peroxidase (HRP)-conjugated goat anti-rabbit IgG and goat anti-mouse IgG secondary antibody (Thermo Fisher).

### Immunoblotting.

The cells were lysed, and proteins were separated by SDS-PAGE and Western blotting was performed as previously described (19). The membrane was incubated with primary and HRP-conjugated secondary antibodies (Pierce). Detection was performed by using a SuperSignal West Pico or Femto kit (Pierce). For semiquantitative analysis, the Gel Analyzer program in Fiji/Image J was used.

### Immunofluorescence.

Cells were grown on coverslips to 20 to 40% confluence, transfected, and incubated for 24 h. Cells were washed, fixed, and stained with antibodies. Confocal images were acquired using a Nikon A1R laser scanning confocal microscope (Nikon) with a 60× oil immersion lens (Plan-Apochromat VC) under the control of NIS-Elements microscope imaging software (Nikon). For live-cell microscopy, 140,000 HeLa cells were seeded in a 35-mm MatTek glass-bottom dish. Cells were transiently transfected with eGFP-GBF1 and mCherry-viperin using Lipofectamine 2000 (Invitrogen) according to the manufacturer's recommendations. After 8 h, the transfection medium was replaced with fresh medium, followed by incubation for another 16 h. Spinning-disc confocal live-cell microscopy was performed at 5% CO_2_ using a 63× objective lens (Plan-Apochromat 1.40 Oil DIC M27) in a Cell Observer spinning disc confocal microscope system (Andor iXon Ultra; Zeiss) controlled by ZEN software. Image analysis and preparation were completed using ImageJ and Adobe Photoshop CS5.

### Viral infection, quantification, and titration.

LGTV infection and viral titers were determined by a focus-forming assay as previously described (41). Total RNA was isolated at 48 h postinfection using NucleoSpin RNA II kit (Macherey-Nagel) as previously described (41), and cDNA was synthesized from 500 ng of RNA using a QuantiTect reverse transcription kit (Qiagen) according to the manufacturer's instructions. mRNA expression of actin was detected by a QuantiTect primer assay (Qiagen) and the Kapa SYBR FAST qPCR kit (Kapa Biosystems) using a StepOnePlus fast-real-time PCR system (Applied Biosystems). TBEV RNA was quantified using previously described primers (57) and a Kapa Probe Fast qPCR kit (Kapa Biosystems).

### Concentration, purification, and flotation of VLPs and virus particles.

Supernatants of transfected or infected cells were collected, concentrated by ultracentrifugation as previously described (58), and resuspended in reducing Laemmli SDS-PAGE sample buffer before Western blot analysis. In the case of further analysis steps, particles were concentrated by ultracentrifugation through a 20/60% sucrose (sucrose in TN buffer [0.1 M NaCl, 0.05 M Tris-HCl; pH 7.4]) cushion at 100,000 × *g* for 1.5 h at 4°C (SW32; Beckman Coulter), and the interface between 20 and 60% sucrose was harvested. A flotation assay was performed as previously described (59). Briefly, the sucrose concentration was adjusted to a final concentration of ∼60% sucrose, incubated with or without 1% Triton X-100 for 1 h at 4°C, overlaid with a 30 and 10% sucrose solution, and centrifuged at 200,000 × *g* for 5 h at 4°C (SW60; Beckman Coulter). Fractions were collected and further concentrated by ultracentrifugation (100,000 × *g*, 45 min, 4°C, SW41; Beckman Coulter).

### Electron microscopy.

Supernatants from cells transfected with C-FLAG or the structural proteins prM and E or infected with LGTV were harvested and concentrated using a flotation assay. Glow-discharged Formvar carbon-coated nickel grids were floated on drops of the virus or VLP suspensions washed with water, fixed with glutaraldehyde 2.5% for 2 min, washed again with water, and stained with 1.5% aqueous uranyl acetate.

### Affinity purification/LC-MS/MS experiments.

293 FLP-IN T-Rex cells expressing FLAG-tagged viperin, Δ1-50 mutant, or green fluorescent protein (GFP) were lysed, and α-FLAG antibody coupled beads were used to immunoprecipitate proteins of interest. Four independent affinity purifications were performed for each bait. Sample preparations and LC-MS/MS analysis were conducted as described previously (60). Briefly, FLP-IN cells expressing the FLAG-tagged protein of interest were lysed by snap-freezing cells in liquid nitrogen, incubation in TAP buffer (50 mM Tris [pH 7.5], 100 mM NaCl, 5% [vol/vol] glycerol, 0.2% [vol/vol] Nonidet P-40, 1.5 mM MgCl_2_, and protease inhibitor cocktail [EDTA-free, cOmplete; Roche]) for 30 min on ice, and clarification of the lysate by centrifugation at 16,000 × *g*. α-FLAG antibody-coupled beads were incubated with 6-mg portions of cleared lysates for 60 min on a rotating wheel, and the proteins were precipitated and washed with TAP buffer. After the final three washes in TAP buffer, the samples were in also washed twice with TAP buffer lacking Nonidet P-40 to remove residual detergent. Samples were sequentially digested with LysC (Wako Chemicals USA) and trypsin (Promega), acidified with 0.1% TFA, desalted with C18-stage tips, and analyzed by LC-MS on an Orbitrap XL instrument (Thermo Fisher Scientific).

Mass spectrometry raw files were processed with MaxQuant software versions 1.5.1.1 (61, 62) using the built-in Andromeda engine to search against human and mouse proteomes (UniprotKB, release 2012_06) containing forward and reverse sequences. In MaxQuant, the label-free quantitation (LFQ) (63) algorithm and the Match Between Runs option were used as described previously (60). The MaxQuant output tables were transferred to the Perseus computational platform (64) for statistical enrichment analysis. Only proteins identified on the basis of at least two peptides and a minimum of three quantitation events in at least one experimental group were considered. LFQ protein intensity values were log transformed, and missing values were supplied by imputation. Specific enrichment was determined by multiple equal variance *t* tests with permutation-based false discovery rate (FDR) statistics (*n* = 250 permutations). FDR thresholds and S0 parameters were empirically set to separate background from specifically enriched proteins.

### Coimmunoprecipitation.

HEK293T cells were transfected 24 h before cell lysis (1 mM MgCl_2_, 1 M Tris-HCl, 5 M NaCl, 5% glycerol, 0.2% NP-40, and protease inhibitor). The FLAG-viperin/GBF1 complex was immunoprecipitated with monoclonal antibodies directed against FLAG (Stratagene), as previously described (19, 65).

### CRISPR Cas9 knockdown of GBF1.

Three different targets for human GBF1 were selected and cloned into pSpCas9(BB)-2A-GFP (PX458) (Addgene number 48138) using the protocol established by F. Zhang (66). The primer sequences were as follows: TBE 434F, CRISPR Cas9 GBF1.1 (CACCGATGGATTACGTCAATCCCCG); TBE 435R, CRISPR Cas9 GBF1.1 (AAACCGGGGATTGACGTAATCCATC); TBE 436F, CRISPR Cas9 GBF1.2 (CACCGACACGACCGCCATAACTCAG); TBE 437R, CRISPR Cas9 GBF1.2 (AAACCTGAGTTATGGCGGTCGTGTC); TBE 438F, CRISPR Cas9 GBF1.3 (CACCGACAGTGATTGACAGCACCG); and TBE 439R, CRISPR Cas9 GBF1.3 (AAACCGGTGCTGTCAATCACTGTC). All three target plasmids were cotransfected at a ratio of 1:1:1; at 48 h posttransfection, the cells were reseeded and used for experiments.

## Supplementary Material

Supplemental material
