# Supplementary material for: Viperin Targets Flavivirus Virulence by Inducing Assembly of Noninfectious Capsid Particles
Source: J Virol. 2017 Dec 14;92(1):e01751-17. doi: 10.1128/JVI.01751-17 (PMC5730767; doi:10.1128/JVI.01751-17)
Supplement: Supplemental material [file supp_92_1_e01751-17__index.html]

Viperin Targets Flavivirus Virulence by Inducing Assembly of Noninfectious Capsid Particles — Supplemental material 

# Viperin Targets Flavivirus Virulence by Inducing Assembly of Noninfectious Capsid Particles

## Supplemental material

- Supplemental file 1 -

  Table S1 (Proteins specifically binding to viperin or the Δ1-50 mutant.)

  XLSX, 15K
